# Complementary medicine in Germany: a multi-centre cross-sectional survey on the usage by and the needs of patients hospitalized in university medical centers

**DOI:** 10.1186/s12906-021-03460-6

**Published:** 2021-11-23

**Authors:** Ann-Kathrin Lederer, Alexandra Baginski, Lena Raab, Stefanie Joos, Jan Valentini, Carina Klocke, Yvonne Samstag, Katrin Hübner, Ivana Andreeva, Thomas Simmet, Tatiana Syrovets, Susanne Hafner, Anna Freisinger, Maximilian Andreas Storz, Roman Huber

**Affiliations:** 1 Center for Complementary Medicine, Department of Medicine II, Medical Center – University of Freiburg, Faculty of Medicine, University of Freiburg, Theodor von Frerichs Haus, Sir-Hans-A.-Krebs-Straße, 79106 Freiburg, Germany; 2 Chirurgische Klinik, Evangelisches Diakoniekrankenhaus, Freiburg, Germany; 3 Institute for General Practice and Interprofessional Care, University Hospital Tübingen, Tübingen, Germany; 4 Institute of Immunology, Section Molecular Immunology, University of Heidelberg, Heidelberg, Germany; 5 Institute of Pharmacology of Natural Products & Clinical Pharmacology, Ulm University, Ulm, Germany

**Keywords:** Complementary therapies, Surveys and questionnaires, Health knowledge, Attitudes, Practice

## Abstract

**Background:**

The results of recent surveys indicate that more than 50% of the German population has experience with complementary and alternative medicine (CAM) or uses CAM regularly. This study investigated the CAM usage and CAM-related needs of hospitalized patients at university medical centres in the state of Baden-Württemberg, Germany.

**Methods:**

A multi-centre, paper-based, pseudonymous survey was carried out by the members of the Academic Centre for Complementary and Integrative Medicine. Patients of all ages, regardless of sex, diagnosis and treatment, who were hospitalized in the Department of Cardiology, Gastroenterology, Oncology, Gynaecology or Surgery at the university medical centres in Freiburg, Heidelberg, Tübingen and Ulm were eligible for inclusion.

**Results:**

Of the 1275 eligible patients, 67% (*n* = 854) consented to participate in the survey. Forty-eight percent of the study participants stated that they were currently using CAM. The most frequently used therapies were exercise (63%), herbal medicine (54%) and dietary supplements (53%). Only 16% of the patients discussed CAM usage with their attending physician. Half of the patients (48%) were interested in CAM consultations. More than 80% of the patients desired reliable CAM information and stated that physicians should be better informed about CAM.

**Conclusions:**

The frequency of CAM usage and the need for CAM counselling among hospitalized patients at university medical centres in Baden-Württemberg are high. To better meet patients’ needs, CAM research and physician education should be intensified.

**Trial registration:**

German Clinical Trial register (DRKS00015445).

## Background

Complementary and alternative medicine (CAM) is commonly used in Europe. According to the results of a European survey published in 2018, every fourth European citizen reported using CAM medicine during the past 12 months before the survey [1]. The usage of CAM was even more frequent in German-speaking countries, with up to 70% of the German population having experience with CAM [2]. CAM comprises non-mainstream therapeutic approaches, such as anthroposophical medicine, acupuncture, traditional Chinese medicine, homeopathy and herbal medicine. CAM is often used without the prior consultation of a physician [2, 3]. Research suggests that there is limited communication about CAM between medical staff and patients [4, 5]. Publications in recent decades reported that every second to third hospitalized patient used CAM, but less than a third of these patients communicated about their CAM usage with their attending physicians [4, 6–10]. The gap between the frequency of CAM usage and communication between patients and physicians emphasizes the need for improvements in medical education about CAM. In 2016, the Academic Centre for Complementary and Integrative Medicine (AZKIM) was founded to promote CAM education and CAM research in Germany. AZKIM is a cooperative centre involving the university medical centres in Freiburg, Heidelberg, Tübingen and Ulm that is financed by the Ministry of Science, Research and Arts of the state of Baden-Württemberg, Germany. The aim of AZKIM is to provide a well-founded knowledge base regarding CAM and integrative medicine for use in basic and clinical research, patient care and training and the continuing education of physicians and medical students. Previous publications showed that many patients desire CAM counselling and physicians who are knowledgeable about CAM, even during a hospital stay, especially as these aspects pertain to a patient-centred and holistic treatment approach [4, 9, 11]. Little is known about CAM use by patients hospitalized in university hospitals. The primary aim of this study was to identify the frequency of CAM usage, the attitudes towards and interest in CAM and the need for CAM counselling among patients hospitalized at the four university medical centres in Freiburg, Heidelberg, Tübingen and Ulm.

## Methods

Between April and December 2018, a multicentre, paper-based, multidisciplinary, pseudonymous cross-sectional study was carried out at the German university medical centres in Freiburg, Heidelberg, Tübingen and Ulm. Patients of all ages, regardless of sex, diagnosis and treatment, who were hospitalized in the Department of Cardiology, Gastroenterology, Oncology, Gynaecology, or Surgery at the university medical centres in Freiburg, Heidelberg, Tübingen and Ulm were eligible for inclusion. Patients had to be able to give their written informed consent before inclusion, to complete the questionnaire on their own and to speak and understand German (at least level B2 according to the European Framework of Reference for Languages [12]). Outpatients and patients in the intensive care unit were excluded.

The study was registered at the German Clinical Trial register (DRKS00015445) and approved by the ethics committee of the University Medical Centre of Freiburg, Germany (EK FR 25/17), before the study was initiated.

### Survey

The questionnaire contained 15 main questions and 6 subordinate questions covering socio-demographic variables such as age, sex and education level; diagnosis; and current quality of life (on a scale from 1 to 10). Additional questions focused on current and previous usage of 21 different CAM approaches (Table 1), knowledge about CAM (“How informed do you consider yourself to be about CAM?”- Patients had to choose between well-informed/informed/poor informed/ not-informed/indecisive; further questions asked for source of informations), interest in CAM (“Are you interested in CAM consultation regarding your current disease?”- agree or disagree), financing of CAM (“How did you finance CAM usage?” and “How much money did you spend for CAM usage?”) and communication about CAM usage (“Do you communicate CAM usage to your attending physician?” – yes or no; further questions asking for reasons and in case of communication, for reaction of the attending physician). In case of CAM usage, motivation for CAM usage (e. g. “I did this/I am going to do this because I want to increase my well-being” –agree or disagree), and in case of non-usage, reasons for non-usage were captured (e. g. “I did not use CAM because I did not need it” – agree or disagree). Furthermore, patients were asked to state their expectations of the attending physicians (e. g. “It is important to me that my attending physician is informed about CAM” – agree or disagree) and their treatment needs (e. g. “It is important to me that I am treated in a holistic way” – agree or disagree).

**Table 1:** CAM approaches considered in the questionnaire

CAM approaches	
Acupuncture/acupressure	Mental Healing, Mindfulness
Anthroposophical medicine	Homeopathy
Aroma therapy	Hyperthermia
Detoxification	Mistletoe treatment
Ayurveda	Osteopathy, Chiropractic
Balneotherapy	Herbal medicine
Exercise	Traditional Chinese Medicine (TCM)
Colonic cleansing, Probiotics	Dietary supplements
Diet & Nutrition	Compresses
Relaxing, Mediation	Yoga, Qigong
Fasting	

CAM usage was assessed by the following question: “Have you ever used or are you still using at least one of the aforementioned CAM approaches (Table 1) for your current disease?”. The need for CAM counselling was assessed by the following question: “Would you like to receive CAM counselling regarding your current disease?”

The questionnaire was modified from two questionnaires, which were previously used by the authors [13, 14], and required approximately 30 min for completion. It was only available in German. The descriptive parameters included age, sex, education level, nationality (German - yes/no), department of hospitalization (cardiology, gastroenterology, oncology, gynaecology or surgery), location of hospital (Freiburg, Heidelberg, Tübingen, Ulm), and oncological or non-oncological treatment. These parameters were also considered factors potentially associated with CAM usage and the need for counselling.

### Management of bias

Patients were asked to answer the questionnaire independently to avoid being influenced by others. Incomplete or formally incorrect (multiple answers for single answer questions) questionnaires were detected by the study staff. Questions were formulated in a neutral manner, and leading questions were avoided. The study staff was encouraged to appear friendly but non-committal to avoid the generation of response tendencies. To prevent language barriers, speaking and understanding German was defined as an inclusion criterion. Recruitment was carried out consecutively, and the questionnaire’s topic was not explained in detail before starting.

### Statistics

The sample size needed for multiple regression was calculated. Based on previous publications, a sample size of at least 140 patients was needed for a reliable multiple logistic regression including 7 predictive variables (age, older vs. younger patients, male vs. female patients, department of hospitalization, survey location, nationality and oncologic disease) [15, 16]. As a response rate of 60% is recommended for validity, we determined that we needed a sample size of at least 250 patients per location (a total of 1000 patients at 4 locations) [15]. Upon survey completion, data were transferred to a pre-designed table (Microsoft Excel) by three authors. IBM SPSS (Version 27.0) was used for the evaluation. The chi-square test was used to analyse the distribution and compare the characteristics of all analysed categorical variables. *P* < 0.05 was considered significant. Unless otherwise stated, the results are given as the percentage of patients who answered a question or as absolute values. Missing data were not imputed.

## Results

**Fig. 1:**
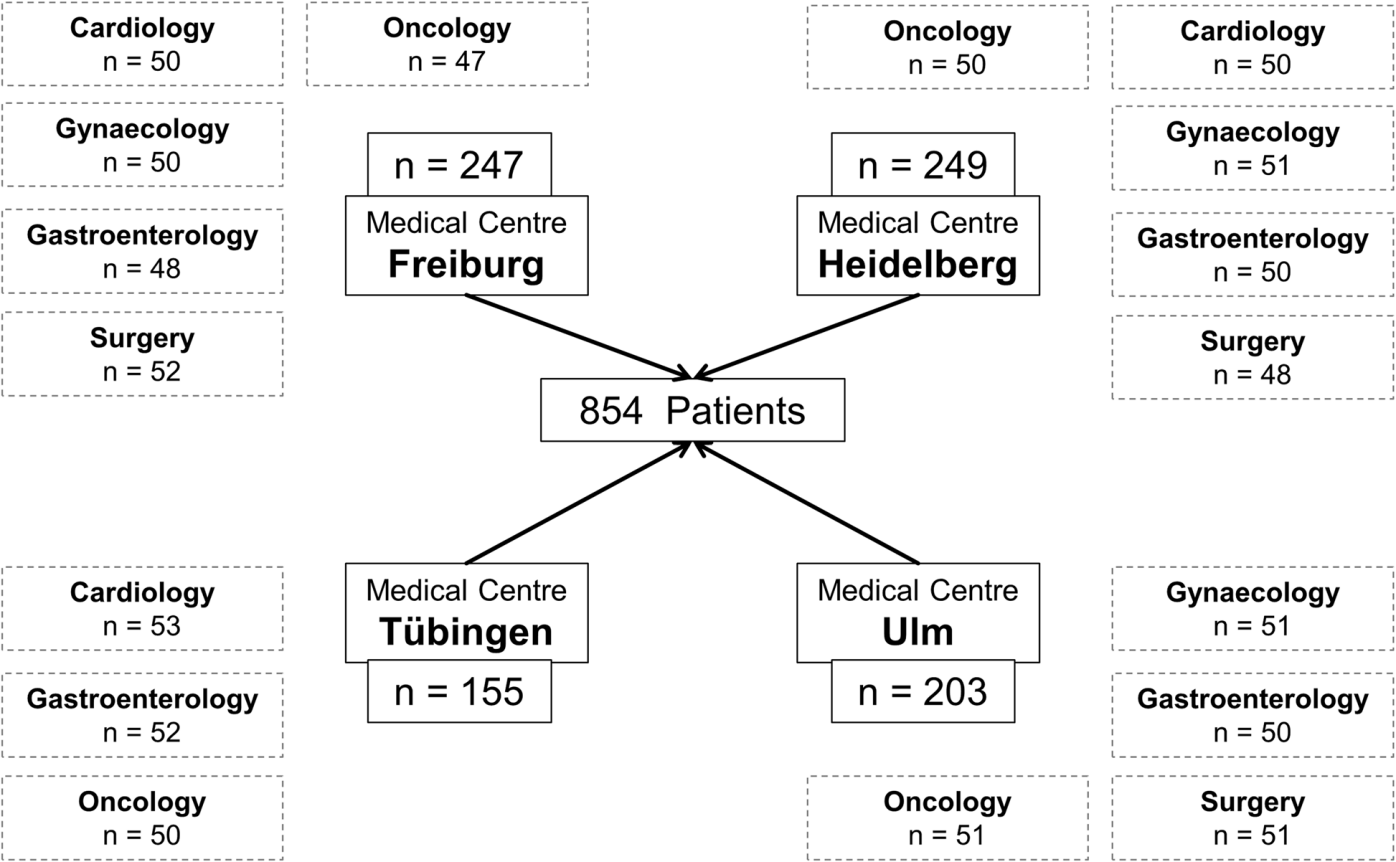
Flowchart of included patients. In Tübingen, no gynaecological and surgical patients were included for organizational reasons. In Ulm, no cardiologic patients were included for organizational reasons

A total of 1275 patients were eligible for inclusion. Two-thirds of these patients (*n* = 854 patients, 67%) consented to participate in the survey. Participating patients were treated in the Department of Cardiology (18%), of Surgery (18%), of Gynaecology (18%), of Oncology (23%) and of Gastroenterology (23%) (Fig. 1 and Table 2). The mean age of the patients was 58 years (range 15–88 years), and 50% of patients were female. Further descriptive patient data are shown in Table 2.

**Table 2:** Overview of descriptive patient data (*n* = 854). All values were rounded to whole numbers

	n	%*	Not clarified (n)
*Department of treatment*			
Cardiology	153	18%	
Gastroenterology	200	23%	
Gynaecology	152	18%	0
Oncology	198	23%	
Surgery	151	18%	
*Sex*			
Female	417	50%	13
Male	424	50%	
*Education level*			
No graduation	15	2%	35
Short-term secondary school (“Hauptschule”)	192	23%	
Elementary school (“Volksschule”)	30	4%	
Vocational school (“Berufsschule”)	12	2%	
School for handicapped children	1	0%	
Intermediate-term secondary high school (“Mittlere Reife”)	256	31%	
High school (“Abitur”)	105	13%	
University	208	25%	
*Nationality*			
German	787	94%	16
Italian	9	1%	
Turkish	7	1%	
Romanian	3	1%	
Other	32	3%	
> 60 years of age	439	52%	15
< 60 years of age	400	48%	
Cancer	332	39%	5

(*percentage of all patients who answered the question)

### Current or previous usage of CAM

A total of 372 of 777 patients (48%) stated that they used CAM currently or had used CAM previously for their current disease (cardiology: 39%, surgery: 44%, gynaecology: 47%, oncology: 52%, gastroenterology: 54%). CAM usage depended on the survey location (Freiburg: 56%, Heidelberg: 51%, Tübingen: 46%, Ulm: 36%) and nationality (German: 49%, other: 28%) and department of hospitalization (cardiology: 38%, gastroenterology: 54%, oncology: 52%, gynaecology: 47%, surgery: 44%, Table 3). Further factors, which might affect current or previous usage of CAM, are shown in Table 3.

**Table 3:** Factors affecting answering the question “Have you ever used before or are you still using at least one of the aforementioned CAM approaches regarding your current disease?”

Parameter	Distribution	Regression coefficient	Standard error	p	Odds ratio	95%-Confidence Interval	
						Lower	Upper
Age	–	0.011	0.009	0.208	1.011	0.994	1.028
< 60 years of age	49%	−0.079	0.260	0.761	0.924	0.555	1.538
> 60 years of age	51%	Reference	Reference	Reference	Reference	Reference	Reference
*Sex*							
Male	49%	0.249	0.173	0.149	1.283	0.915	1.799
Female	51%	Reference	Reference	Reference	Reference	Reference	Reference
*Department*							
Cardiology	17%	0.740	0.254	**0.004**	2.095	1.273	3.450
Gastroenterology	23%	Reference	Reference	Reference	Reference	Reference	Reference
Gynaecology	19%	0.511	0.271	0.059	1.667	0.981	2.833
Oncology	24%	0.130	0.245	0.595	1.139	0.704	1.843
Surgery	17%	0.435	0.252	0.084	1.545	0.943	2.530
*Survey location*							
Freiburg	31%	−0.302	0.200	0.130	0.739	0.500	1.093
Heidelberg	27%	Reference	Reference	Reference	Reference	Reference	Reference
Tübingen	16%	0.218	0.249	0.383	1.243	0.763	2.027
Ulm	26%	0.640	0.216	**0.003**	1.896	1.241	2.898
*Cancer*							
Yes	40%	−0.007	0.191	0.969	0.993	0.683	1.1443
No	60%	Reference	Reference	Reference	Reference	Reference	Reference
*Nationality*							
German	94%	0.915	0.347	**0.008**	2.496	1.264	4.927
Other	6%	Reference	Reference	Reference	Reference	Reference	Reference

Multiple logistic regression (Nagelkerke *R*^*2*^ = 0.084; H = 0.336), highest sample-size group was chosen as reference. Only patients for whom a complete data set was available were evaluated (*n* = 762). Patients in Heidelberg used CAM more frequently than patients from Ulm (51 vs. 36%). The CAM usage was significantly more frequent in patients of German nationality than in patients of other nationalities (49 vs. 28%). Patients hospitalized at the department of gastroenterology had a higher CAM usage frequency than cardiologic patients (54 vs. 39%).

**Fig. 2:**
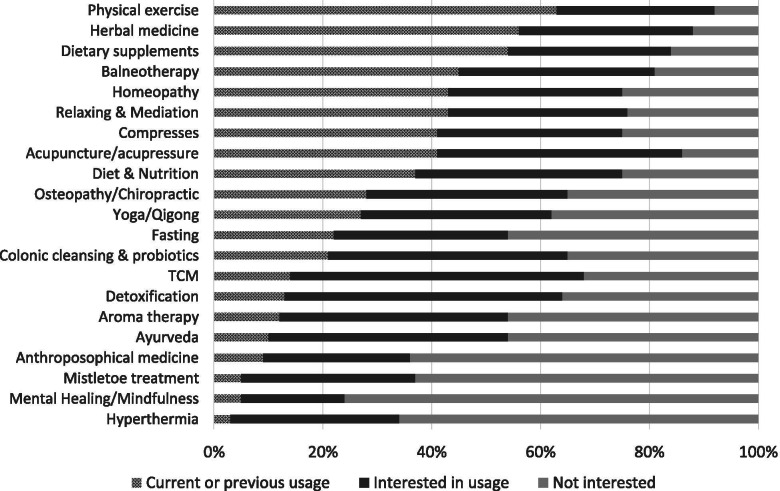
Current and previous usage of various complementary medicine approaches as well as interest in treatment. (TCM = Traditional Chinese Medicine)

From the list of 21 CAM approaches (Table 1), exercise (63% of 834 patients), herbal medicine (56% of 843 patients), intake of dietary supplements (54% of 834 patients), balneotherapy (45% of 824 patients), relaxation therapy (43% of 840 patients) and homeopathy (43% of 835 patients) were the most frequently used (Fig. 2).

Patients who stated that they currently used CAM were asked about their reasons for using CAM. Patients stated that they used CAM to increase their well-being (87% of 437 patients), to positively affect their health (86% of 426 patients) or to actively treat their disease (86% of 437 patients). CAM usage was motivated by a favourable attitude towards holistic therapy in almost 60% of the 435 responding patients. One-third of the patients (29% of 430 patients) stated that the lack of success of conventional therapy was the reason for their usage of CAM.

Overall, 405 of 777 patients (52%) reported that they did not currently use CAM. The most common reasons given were the lack of CAM counselling (50%), lack of reliable CAM information (42%) and no need for CAM (42%). One-third of these patients (32%) questioned the efficacy of CAM, and another one-third (34%) feared interactions with their conventional therapy. Only a few patients indicated that attending physicians (7%) or friends (6%) discouraged them from using CAM.

### Demands and expectations of attending physicians

A total of 382 patients (48%) out of the 795 responding patients desired CAM counselling for their current disease (responded “yes” when they were asked about their need for CAM counselling). Furthermore, 225 patients (26%) stated “I don’t know”, and 188 patients (24%) stated they did not need CAM counselling. The results of multiple logistic regression are shown in Table 4.

**Table 4:** Factors affecting answering the question “Would you like to have CAM counselling regarding your current disease?”

Parameter	Distribution	Regression coefficient	Standard error	p	Odds ratio	95%-Confidence Interval	
						Lower	Lower
Age	–	0.009	0.011	0.383	1.010	0.988	1.031
< 60 years of age	49%	−0.289	0.325	0.787	0.749	0.396	1.418
> 60 years of age	51%	Reference	Reference	Reference	Reference	Reference	Reference
*Sex*							
Male	47%	0.612	0.218	**0.005**	1.845	1.204	2.827
Female	53%	Reference	Reference	Reference	Reference	Reference	Reference
*Department*							
Cardiology	16%	0.910	0.316	**0.004**	2.483	1.337	4.613
Gastroenterology	24%	Reference	Reference	Reference	Reference	Reference	Reference
Gynaecology	20%	0.659	0.350	0.059	1.933	0.947	3.838
Oncology	21%	0.250	0.328	0.446	1.285	0.675	2.445
Surgery	19%	0.277	0.300	0.357	1.319	0.732	2.376
*Survey location*							
Freiburg	29%	0.608	0.265	**0.022**	1.837	1.093	3.088
Heidelberg	28%	Reference	Reference	Reference	Reference	Reference	Reference
Tübingen	15%	0.471	0.329	0.152	1.602	0.840	3.054
Ulm	28%	0.981	0.278	**< 0.001**	2.668	1.548	4.598
*Cancer*							
Yes	39%	−0.358	0.245	0.144	0.699	0.432	1.130
No	61%	Reference	Reference	Reference	Reference	Reference	Reference
*Nationality*							
German	93%	0.311	0.348	0.373	1.364	0.689	2.701
Other	7%	Reference	Reference	Reference	Reference	Reference	Reference

Multiple logistic regression (Nagelkerke *R*^*2*^ = 0.121; H = 0.046), highest sample-size group was chosen as reference. Only patients for whom a complete data set was available and who answered “yes” or “no” were evaluated (*n* = 562). Female patients stated a higher need for CAM counselling (73 vs. 60%). Patients hospitalized at the department of gastroenterology had a higher need for CAM counselling than cardiologic patients (72 vs. 51%). Patients in Heidelberg stated a higher need for CAM counselling (77%) than patients in Freiburg (64%) and Ulm (60%).

The need for reliable CAM information was reported by more than 80% of the 814 responding patients. Nearly 80% of these patients stated that attending physicians should be informed about CAM. University CAM research was desired by nearly 80% of the 816 responding patients. More than half of the patients felt poorly informed (39% of 781 patients) or completely uninformed about CAM (21%). Sources of information about CAM are shown in Table 5.

**Table 5:** Overview of CAM information sources for all patients (*n* = 854)

Information source	Percentage of positive responses (n)
General practitioner	47% (404)
Medical specialists	25% (214)
Clinicians	16% (136)
Pharmacists	16% (135)
Non-medical practitioners	13% (113)
Nurses	7% (56)
Internet	46% (393)
Brochures	29% (251)
Books	22% (187)
Magazines	21% (178)
Conferences	3% (29)
Friends and family	23% (200)
Self-help group	7% (56)

Only 16% of the total of 823 responding patients stated that they had communicated their interest in or use of CAM to their attending physician. More than one-third of the patients (39%, *n* = 45) who had communicated about CAM stated that their physicians supported their use of CAM, and 30% of the patients reported that CAM was recommended by the attending physicians. Only 16% (*n* = 18) reported that their physicians had a negative attitude towards CAM, and 33% (*n* = 38) questioned the efficacy of CAM. Among the patients who did not communicate about CAM with their attending physician (*n* = 689), 26% stated (*n* = 116) that the reason was that they were afraid of a negative response from their physician. Half of the responding patients (51%, *n* = 250) indicated that they did not have time for a conversation about CAM.

### Financing of CAM

A total of 428 patients gave information about CAM financing. A total of 72% paid for CAM themselves. Only 20% of the patients stated that their statutory health insurance had covered the CAM costs. An average amount of 1220 ± 2857 € was spent on current CAM treatment. The high cost was stated as the reason for not using CAM by 22% of patients who did not use CAM. On average, more than 80% of the 819 responding patients wanted the cost of CAM to be covered by statutory health insurance.

## Discussion

The results of our survey emphasize the relevance of CAM in the context of university medical centre inpatient treatment, as nearly 50% of the patients were using CAM. The most frequently used methods were physical exercise, herbal medication and dietary supplements. Furthermore, nearly half of the patients were clearly interested in CAM counselling and thought that physicians should be informed about CAM.

### Limitations and strengths

The strength of our survey was the response rate of 67%, as a response rate of approximately 60% is recommended based on recent research to avoid bias [15]. However, selection bias could not be ruled out since it is could be assumed that patients interested in CAM are also more interested in and compliant with participating in a CAM-related survey. The study was only carried out in Baden-Württemberg, implying limited generalizability of the results to other regions in Germany as well as to other countries all over the world.

### Selection of patients by CAM financing and comparison with CAM usage in other surveys

Baden-Württemberg is one of the wealthiest regions in Germany [16]. More than two-thirds of the patients financed CAM privately, and CAM treatments are expensive. Thus, many patients with limited means are probably not able to afford CAM. A comparable survey performed in 2018 at the medical centre in Chemnitz (a non-university maximum-care hospital in Eastern Germany) by our research group showed a lower frequency of current CAM usage of 30% in orthopaedic and trauma patients [4]. Chemnitz is a low-income region compared to Baden-Württemberg. Reimbursement for CAM has been discussed for decades in medical journals [17–19]. In 2004, most herbal medications were removed from the statutory health insurance catalogue of reimbursable medications in Germany for financial reasons. In Switzerland, the CAM costs incurred by 60% of the population are covered by additional insurance [20]. The majority of patients in our survey stated that effective CAM approaches should be covered by health insurance, which was also reported in Chemnitz and is confirmed by the high proportion of patients with additional insurance in Switzerland. Nevertheless, the interest in CAM and usage of CAM were similar in the study performed in Chemnitz and this survey, emphasizing the high level of interest in CAM among patients in Germany. A survey of inpatient internal medicine patients carried out in Switzerland in 2014 showed that 32% had used CAM during or 2 months prior to hospitalization, and herbal medicine was the most common modality [7]. Similarly, Schieman et al. found a preference for herbal medicine, with an overall frequency of CAM usage of 27% in surgical patients in Canada [6]. In addition, dietary supplements were frequently used, which is in accordance with the results of our study and the study performed in Chemnitz [4]. Teo et al. reported a CAM usage frequency of 44% in hospitalized patients with cardiovascular diseases in Singapore in 2016 [8]. The CAM approaches in that study (TCM and Jamu, a traditional Indonesian herbal medicine) differed from ours, which can be explained by cultural differences, but again, herbal medication was the predominant modality. Other German surveys including non-hospitalized patients with chronic illnesses such as inflammatory bowel diseases, multiple sclerosis or cancer reported a CAM usage frequency of up to 72%, with the predominant use of dietary supplements and herbal medicine [21–25].

### Non-communication about CAM

Notably, only a minority (16%) of patients stated communicating about CAM with their attending physician. Other recent studies reported that up to one-third of patients communicated about their CAM usage with their attending physician. Communication differs between inpatients and outpatients and depends on the attending physician’s specialization [4, 9, 11, 26–28]. Kilper et al. reported that only 15% of inpatient orthopaedic and trauma patients in Germany indicated their use of CAM [4]. In a Hungarian study, 13% of inpatient surgical patients told their attending physician about their CAM interest [9]. A further survey in Israel showed that only 12% of patients communicated about CAM with clinicians, whereas a significantly larger proportion (50%) communicated with general practitioners [29]. In an American survey of patients undergoing radiation, only 12% of patients stated a conversation about CAM usage with their radiologist [30]. The results of a German online survey of an oncological self-help group showed that 28, 24 and 10% of the patients communicated about CAM with oncologists, general practitioners and other physicians, respectively [11]. The lack of communication poses risks, as potential interactions between herbal medicines or dietary supplements and conventional therapies might be missed. Improvements are needed in communication about CAM.

### The demand for reliable information about CAM and CAM informed physicians

As shown in our study, patients are interested in reliable information about CAM [4]. Patients in our study, as well as in previous publications, felt poorly informed about CAM [22] and wanted to receive combined or holistic treatment with CAM and conventional medicine [2, 4, 23]. More than half of the patients in our trial stated that they had an interest in CAM counselling, and more than 80% wished that attending physicians knew about CAM. This was also reported in other surveys [4, 9–11]. Furthermore, patients want improved medical education and research about CAM. Physicians should be informed about CAM, not only to improve patient safety but also to ensure quality and promote patient-centred treatment.

## Conclusion

This study shows the relevance of CAM at university medical centres in the state of Baden-Württemberg, Germany. The frequency of CAM usage was high. Furthermore, the results of our study emphasize a high level of the need for CAM counselling and desire for the attending physicians to be informed about CAM. Communication about CAM is still poor, indicating the need to actively ask patients about their CAM usage. To facilitate patient-centred treatment and ensure treatment safety and quality, physicians should be better informed about CAM.

## Data Availability

The datasets used and analysed during the current study are available from the corresponding author on reasonable request.

## Abbreviations

AZKIM: Akademisches zentrum komplementäre und integrative medizin (academic center for complementary and integrative medicine)
CAM: Complementary medicine
TCM: Traditional Chinese medicine
